# Increased HRD score in cisplatin resistant penile cancer cells

**DOI:** 10.1186/s12885-022-10432-7

**Published:** 2022-12-23

**Authors:** Ria Winkelmann, Katrin Bankov, Claudia Döring, Jaroslav Cinatl, Sebastian Grothe, Florian Rothweiler, Martin Michaelis, Christina Schmitt, Peter J. Wild, Melanie Demes, Jindrich Cinatl, Stefan Vallo

**Affiliations:** 1 Dr. Senckenberg Institute of Pathology, University Hospital Frankfurt, Frankfurt Am Main, Germany; 2 Dr. Petra Joh Forschungshaus, Frankfurt Am Main, Germany; 3 Institute of Medical Virology, University Hospital Frankfurt, Frankfurt Am Main, Germany; 4 School of Biosciences, University of Kent, Canterbury, UK; 5 Frankfurt Institute for Advanced Studies (FIAS), Frankfurt Am Main, Germany; 6 Department of Urology, University Hospital Frankfurt, Frankfurt Am Main, Germany; 7 Urologie an der Zeil, Frankfurt Am Main, Germany

**Keywords:** Penile carcinomas, Cell lines, Chemoresistance, Homologous recombination deficiency

## Abstract

**Background/Introduction:**

Penile cancer is a rare disease in demand for new therapeutic options. Frequently used combination chemotherapy with 5 fluorouracil (5-FU) and cisplatin (CDDP) in patients with metastatic penile cancer mostly results in the development of acquired drug resistance. Availability of cell culture models with acquired resistance against standard therapy could help to understand molecular mechanisms underlying chemotherapy resistance and to identify candidate treatments for an efficient second line therapy.

**Methods:**

We generated a cell line from a humanpapilloma virus (HPV) negative penile squamous cell carcinoma (UKF-PEC-1). This cell line was subject to chronic exposure to chemotherapy with CDDP and / or 5-FU to induce acquired resistance in the newly established chemo-resistant sublines (PEC-1^r^CDDP^2500^, adapted to 2500 ng/ml CDDP; UKF-PEC-1^r^5-FU^500^, adapted to 500 ng/ml 5- FU; UKF-PEC1^r^CDDP^2500^/^r^5-FU^500^, adapted to 2500 ng/ml CDDP and 500 ng/ml 5 -FU). Afterwards cell line pellets were formalin-fixed, paraffin embedded and subject to sequencing as well as testing for homologous recombination deficiency (HRD). Additionally, exemplary immunohistochemical stainings for p53 and gammaH2AX were applied for verification purposes. Finally, UKF-PEC-1^r^CDDP^2500^, UKF-PEC-1^r^5-FU^500^, UKF-PEC1^r^CDDP^2500^/^r^5-FU^500^, and UKF-PEC-3 (an alternative penis cancer cell line) were tested for sensitivity to paclitaxel, docetaxel, olaparib, and rucaparib.

**Results and conclusions:**

The chemo-resistant sublines differed in their mutational landscapes. UKF-PEC-1^r^CDDP^2500^ was characterized by an increased HRD score, which is supposed to be associated with increased PARP inhibitor and immune checkpoint inhibitor sensitivity in cancer. However, UKF-PEC-1^r^CDDP^2500^ did not display sensitivity to PARP inhibitors.

**Supplementary Information:**

The online version contains supplementary material available at 10.1186/s12885-022-10432-7.

## Introduction

In developed countries penile cancer is a rare tumor. The incidence in Central Europe and the United States is about 1.0 per 100,000 men per year [1], but the incidence tends to be higher in developing countries. The differences of incidence rates have been attributed to reduced hygiene standards, high rates of sexually transmitted infections and a high rate of uncircumcised men [2]. There are HPV related and non-HPV related types of penile cancer [3]. The non-HPV related penile cancers are for example related to chronic inflammatory precursor lesions, lack of neonatal circumcision and cigarette smoking [4]. The non-HPV related types are anticipated to behave more aggressively compared to HPV associated tumors [5, 6]. If the diagnosis is made early, the chances of recovery are excellent with overall 5-year relative survival rates of 97% for pTis/pTa tumors [1]. However, many patients are diagnosed at an advanced stage [2]. For patients with metastatic disease, prognosis remains extremely poor, with overall 5-year survival rates of less than 5% [1].

In metastasized penile carcinoma, one of the frequently used chemotherapy regimens is a combination of cisplatin (CDDP) and 5-fluorouracil (5-FU) [7–9], but resistance development and subsequent treatment failure are common. Progress on the clinical management of advanced penile cancer has been slow, partly due to the lack of appropriate preclinical models. Recently, there have been reports about new penile cancer cell line models [10–18]. However, the number of (commercially) available cell line models is still low. Here, we established a new penile cancer cell line (UKF-PEC-1). Furthermore, development of acquired cancer cell drug resistance is difficult to study. In this context, drug-adapted cancer cell lines have been successfully used to study acquired drug resistance [19, 20]. Hence, we also established a set of sublines adapted to CDDP, 5-FU, and the combination of CDDP and 5-FU. The cell lines were characterized by sequencing and via OncoScan®Array to determine a homologous recombination repair (HRD) score. Additionally, we tested these cell lines and and an additional penile cancer cell line (UKF-PEC-3, [21]) for sensitivity to paclitaxel, docetaxel, olaparib, and rucaparib.

## Material and methods

### Sample

An unfixed partial penectomy specimen of a 64-year-old patient with a squamous cell carcinoma of the penis measuring 5.4 cm in largest dimension located primarily on the foreskin was received by the Dr. Senckenberg Institute of Pathology, Frankfurt am Main for intraoperative frozen section diagnosis of the resection margins. The tumor was diagnosed as HPV independent penile squamous cell carcinoma, usual type, according to WHO classification of Tumours 5th Edition, Urinary and Male Genital Tumours. The tumor was excised in toto (R0) and showed infiltration of corpus spongiosum without infiltration of the urethra (pT2). Perineural invasion was noted (Pn1). The tumor was graded G2 on a scale of G1 – G3. The tumor showed usual type morphology and infiltrative borders [22]. An adjacent lichen sclerosus was not noted. Additional p16 immunohistochemical staining by CINtec® Histology (Roche Diagnostics, Basel, Switzerland) antibody according to manufacturers’ instructions on Dako Autostainer link 48 (Agilent, Santa Clara, CA, USA) was negative. The patient received additional bilateral inguinal lymphadenectomy showing 28 lymph nodes without tumor infiltration. One year later the patient developed a mediastinal metastasis. Two years after diagnosis of the squamous cell carcinoma the patient deceased. The cause of death is unknown.

### Ethical statement

Formalin fixed and paraffin-embedded (FFPE) material as well as unfixed material was taken from the Dr. Senckenberg Institute of Pathology, University Hospital, Frankfurt am Main, Germany. The material was taken after diagnostics had been finished. It was double pseudonymized. Tissue sample and patient data used in this study were provided by the University Cancer Center Frankfurt (UCT). Written informed consent was obtained from the patient and the study was approved by the institutional review board of the UCT and the ethical committee at the University Hospital Frankfurt, according to the declaration of Helsinki (project-number: SUG-02–2017).

### Cell lines

For primary tumor cell line generation (UKF-PEC-1), a penile cancer specimen (diameter 5 mm) was removed from the primary tumor and dissected with a scalpel into little pieces and treated twice with trypsin (0.2%) for 30 min afterwards. Trypsin was inactivated by 10% FBS. The tumor material was cultivated in epithelial medium (Sciencell, Carlsbad, CA, USA) until cell adhesion. Media was changed at five days intervals. The drug-resistant sublines UKF-PEC-1^r^CDDP^2500^ (adapted to 2500 ng/ml CDDP), UKF-PEC-1^r^5-FU^500^ (adapted to 500 ng/ml 5- FU), and UKF-PEC1^r^CDDP^2500^/^r^5-FU^500^ (adapted to 2500 ng/ml CDDP and 500 ng/ml 5 -FU), were established by continuous exposure to increasing drug concentrations, as described previously [21]. The sublines were derived from the Resistant Cancer Cell Line collection RCCL [23].

In addition, the penile carcinoma cell line UKF-PeC-3 was derived from a HPV DNA subtype 16-positive tumor with a pT3 pN0 L0 G2 R0 [24].

All cell lines were grown in Iscove’s modified Dulbecco’s medium (IMDM) supplemented with 10% fetal calf serum (FCS; Gibco, Thermo Fisher Scientific Inc., USA), 100 IU/ml penicillin and 100 µg/ml streptomycin as well as 4 mM Glutamin at 37 °C. Mycoplasma testing was performed.

To determine population doubling times (PDT, in h), 4 × 10^2^ cells per well were plated into 96-well plates and incubated at 37 °C and 5% CO_2_. After 0, 1, 4 and 7 days, 100 µl of CellTiter-Glo (Promega) was added to each well (according to manufacturer´s instruction) and luminescence was measured on a plate reader (TECAN Spark 20 M, Männedorf, Switzerland). Population doubling times (in h) were then calculated for time points day 1 to day 4 (72 h) using http://www.doubling-time.com/compute.php, which uses the equation: Doubling time = duration × log(2)/log(final cell number) − log(initial cell number). The growth curves were generated using GraphPad Prism 9.3.1.

### Viability assay (MTT Assay)

Cell viability was tested by the 3-(4,5-dimethylthiazol-2-yl)-2,5-diphenyltetrazolium bromide (MTT) dye reduction assay after 120 h of incubation modified as described previously [21]. Cells (2 × 10^4^/100 µL per well in 96-well plates) were incubated in the presence or absence of drug for 120 h. Then, 25 µL of MTT solution (2 mg/mL (*w/v*) in PBS) were added per well, and. plates were incubated at 37 °C and 5 % CO_2_for 4 h. Afterwards, cells were lysed and incubated overnight, using 100 µL of a buffer containing 20% (*w/v*) sodium dodecylsulfate and 50% (*v/v*) N,N-dimethylformamide (pH 4.7) at 37°C and 5 % CO_2_. Absorbance was determined at 560 nm and 620 nm on a plate reader (TECAN Spark 20M, Tecan Group Ltd., Männedorf, Switzerland). After subtraction of the background absorption, the results were expressed as the percentage of viability relative to control cultures that received no drug. Drug concentrations that inhibited cell viability by 50% (IC50) were determined using CalcuSyn (Biosoft, Cambridge, UK).

### Production of a cell block from cell cultures

Cell line cultures were centrifuged and cells pellets were resuspended in phosphate-buffered saline. After addition of formalin and incubation another centrifugation step was added. Thereafter, protein glycerin and ethanol 96% were added followed by centrifugation. Cell pellets were subject to conventional tissue draining machine and processed in normal pathology routine.

### DNA extraction and library preparation

All laboratory work was performed according to manufacturer’s instructions. Representative tumor material of the primary tumor was macrodissected. The cell lines were formalin fixed and paraffin embedded and taken as whole slides. The purification of DNA and RNA from FFPE tissue samples was performed using the Maxwell® RSC Instrument (Promega Corporation, Madison, Wisconsin, USA) with the Maxwell® RSC FFPE Plus RNA Kit (Promega Corporation, Madison, Wisconsin, USA) and QIAamp DNA FFPE Tissue Kit (Qiagen, Hilden, Germany), respectively. The concentration of DNA and RNA was measured with the Qubit 4 Fluorometer (Invitrogen, Thermo Fisher, Waltham, Massachusetts, USA). For the library preparation 20 ng for the OncomineTM Comprehensive Assay v3 (Thermo Fisher, Waltham, Massachusetts, USA) were used. A 540 chip was applied.

### DNA Quality control

For quality assurance the DNA and RNA were analyzed by DNA and RNA Screen Tape assay with the Agilent 4200 TapeStation (Agilent Technologies, Santa Clara, California, USA). This automated electrophoresis solution is used to determine the integrity, too. The RIN and DIN (RNA and DNA Integrity Number) are software algorithms for determining RNA and DNA quality, respectively. RIN and DIN values range from 1 to 10; 10 being completely intact DNA or RNA, 1 being completely degraded. Next-generation sequencing: OncomineTM Comprehensive Assay v3 (Thermo Fisher, Waltham, Massachusetts, USA). Data analysis was performed using the analysis software platforms provided by ThermoFisher. The primary analysis of the sequencing data was completed by Torrent SuiteTM software. Afterwards data were analyzed with the Ion ReporterTM software (version 5.12.0.0), filter chains Oncomine Variants 5.10 and Oncomine Extended 5.12 were used. Genomic alterations were identified by the alignment on the reference genome hg19 (GRCh37) available at www.ncbi.nlm.nih.gov. To achieve reliable results, only alterations with fulfilled quality criteria were considered, such as allele frequency ≥ 5% and a coverage of at least 500 × for the Ion S5TM. Classification and interpretation of detected filtered and unfiltered variants were evaluated. The variant annotation provided by the respective software was manually reviewed according to the online databases ClinVar [25] and Cosmic [26]. Other databases used for validation were: gnomAD, OncoKB, dbSNP and cBioPortal (available online). For this study, the annotation of pathogenicity of the detected variants was determined according to the ClinVar classification in: “benign”, “likely benign”, “uncertain significance”, “likely pathogenic”, “pathogenic”. To achieve a consistent approach of naming all variants, sequence variant nomenclature was carried out in concordance with the guidelines by the Human Genome Variation Society (HGVS).

### Oncoscan array

#### DNA and RNA isolation

For DNA isolation from formalin-fixed, paraffin-embedded (FFPE) patient tumor tissue and FFPE-processed primary parental cells and the adapted chemo-resistant sublines were used. Areas with high tumor content were marked on hematoxylin and eosin (H&E)-stained slides with a 2 μm tissue section by experienced pathologists (RW). Afterwards, tissue cores (1.0 mm diameter) from FFPE blocks were taken from the marked area. DNA was isolated using the truXTRAC FFPE total NA Kit (Covaris, Woburn, MA, USA) based on focused ultrasonification and column purification according to the manufacturer's instructions. The DNA was eluted in 50 μL nuclease-free water and stored at − 20 °C. The DNA was quantified using the Quantus™ Fluorometer and ONE dsDNA Assay Kit (Promega Corporation, Madison, Wisconsin, USA). Additionally, samples have been screened for fragment length using Bioanalyzer 2100 (Agilent Technologies, Santa Clara, California, USA) and Agilent DNA 7500 Kit (Agilent Technologies, Santa Clara, California, USA) in order to include samples with fragments larger than 150 bp for subsequent Copy-number variation analysis.

#### Copy-number variation arrays

Array-based genome-wide copy-number analysis was conducted using OncoScan FFPE Microarrays (Affymetrix, Santa Clara, California, USA). For the assay, an input amount of 79.2 ng DNA was used. The OncoScan® assay utilizes the molecular inversion probe technology (MIP), which is optimized for highly degraded FFPE samples, for the identification of copy number (CN) alterations, loss of heterozygosity (LOH) and somatic mutations (SMs). MIP probes in the OncoScan® assay capture the alleles of over 220,000 SNPs within ~ 900 cancer genes. Furthermore, the MIP probes also enable detection of 74 frequently tested somatic mutations in nine genes implicated in cancer (BRAF, KRAS, EGFR, IDH1, IDH2, PTEN, PIK3CA, NRAS and TP53). The molecular inversion probe processing was done according to the OncoScan FFPE Assay Kit Protocol (Affymetrix, Santa Clara, California, USA). At the end of the hybridization period, arrays were stained and washed using the GeneChip® Fluidics Station 450 and loaded into the GeneChip® Scanner 3000 7G (Affymetrix, Santa Clara, California, USA) where array fluorescence intensity was scanned to generate array images (DAT file). Array fluorescence intensity (CEL) files were automatically generated from DAT files by the Affymetrix® GeneChip® Command Console® (AGCC) Software version 4.0. Resulting data files (CEL files) were processed and viewed by using Chromosome Analysis Suite (ChAS) software version 4.2.0.80. Segmentation was performed using ASCAT (package version 2.5.2, in R version 4.0.5). With this package, we also derived copy-number profiles of tumor cells and estimates of normal cell contamination and ploidy.

#### Analyses of HRD

HRD score was calculated from output of ASCAT using implementations in R as described by Sztupinszki et al. [27]. Estimates of the global levels of LOH, large-scale transition (LST), and telomeric allelic imbalance (TAI) were calculated separately, and the unweighted sum of these was defined as the HRD score. A score of > 42 was used as the cut-off for HRD, as defined in breast cancer [28].

#### Immunohistochemistry

Paraffin sections were stained with the antibodies p53 and gammaH2AX. For staining, the DAKO FLEX-Envision Kit (Agilent, Santa Clara, CA, US) and the fully automated DAKO Omnis staining system or manual procedure (Agilent, Santa Clara, CA, US) were applied according to manufacturer´s instructions. The antibodies used were Anti- Anti-phospho-Histone H2A.X (gammaH2AX) (Ser139) (ZRB05636, dilution 1:100, Merck KgA, Darmstadt, Germany) applied for 30 min after 95 °C epitope retrieval in pH9 and anti-Human p53 Protein (DO-7, ready to use dilution, Dako, Agilent, Santa Clara, CA, US) applied for 20 min after epitope retrieval in pH9. Epitope visualization was performed using DAKO EnVision™ FLEX DAB + Substrate Chromogen System (Agilent, Santa Clara, CA, US). Nuclear counterstain was done using DAKO hematoxylin solution (Agilent, Santa Clara, CA, US). Slides were finally digitized using Pannoramic Scan II (3D Histech, Budapest, Hungary.

## Results

### Resistance profiles

**Fig. 1:**
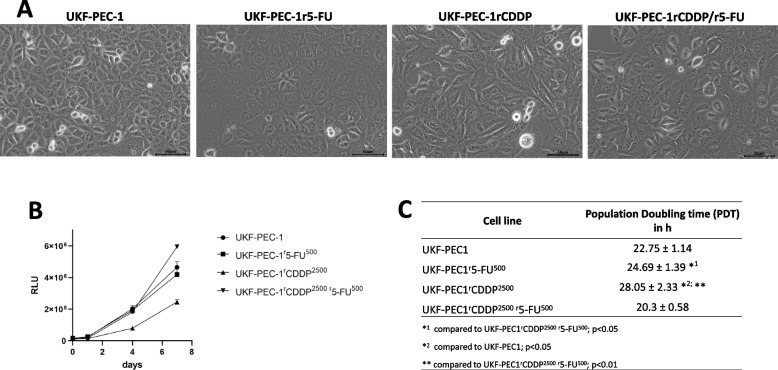
**A** Pictures of cell cultures (20x). **B** Growth curves. **C** Population doubling times (PDT) in h

Figure 1 displays pictures of cell cultures, growth curves and population doubling times (PDT) in h. Resistance was confirmed in the CDDP-adapted (IC50 UKF-PEC1^r^CDDP^2500^: 1.61 μg/mL CDDP ± 0.20 vs. UKF-PEC1: 0.268 μg/mL CDDP ± 0.015) and 5-FU-adapted sublines (IC50 UKF-PEC1^r^5-FU^500^: 1.707 μg/mL 5-FU ± 0.140 vs. IC50 UKF-PEC1: 0.953 μg/mL 5-FU ± 0.042) (Table 1). The double-adapted subline UKF-PEC1^r^CDDP^2500^/^r^5-FU^500^ was resistant against both drugs. UKF-PEC1^r^5-FU^500^ was cross-resistant to treatment with CDDP (Table 1). Drug sensitivity curves are shown in supplementary Fig. 1.

**Table 1:** Concentrations of 5-FU, CDDP, paclitaxel, docetaxel, olaparib and rucaparib that reduce the viability of UKF-PEC-1, UKF-PeC-3 and its drug-adapted sublines by 50% (IC50) as determined by MTT assay after 120 h of incubation

Cell line	IC50 5-FU (µg/ml)	IC50 CDDP (µg/ml)	IC50 paclitaxel (ng/ml)	IC50 docetaxel (ng/ml)	IC50 olaparib (µM)	IC50 rucaparib (µM)
UKF-PEC1	0.953 ± 0.042	0.268 ± 0.015	2.15 ± 1.75	0.567 ± 0.488	8.69 ± 1.45	20.91 ± 3.73
UKF-PEC1^r^5-FU^500^	1.71 ± 0.14	0.623 ± 0.049	2.06 ± 0.54	0.518 ± 0.102	10.07 ± 2.41	16.49 ± 3.62
UKF-PEC1^r^CDDP^2500^	0.13 ± 0.03	1.61 ± 0.2	1.61 ± 1.06	0.465 ± 0.202	15.78 ± 0.89	24.07 ± 0.41
UKF-PEC1^r^CDDP^2500r^5-FU^500^	3.99 ± 0.289	6.66 ± 0.275	1.77 ± 0.59	0.505 ± 0.266	11.61 ± 1.84	22.45 ± 1.38
UKF-PEC3	0.338 ± 0.064	0.943 ± 0.144	2.76 ± 0.18	0.735 ± 0.124	15.79 ± 2.25	34.07 ± 11.39
UKF-PEC3^r^5-FU^1500^	7.83 ± 4.95	2.47 ± 1.37	3.3 ± 0.6	0.913 ± 0.212	32.49 ± 6.39	24.49 ± 1.19
UKF-PEC3^r^CDDP^2000^	0.15 ± 0.016	4.18 ± 0.26	1.47 ± 0.09	0.385 ± 0.026	30.97 ± 6.28	21.59 ± 0.86

**Fig. 2:**
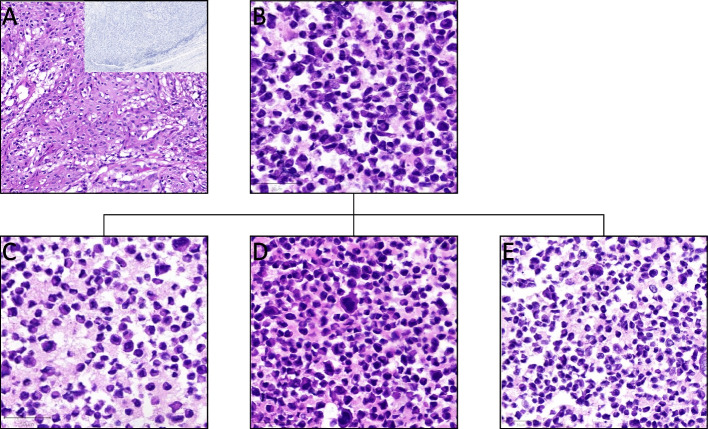
**A**: HE staining of the primary tumor, scale: 50 µm; Inlay: negative p16 staining scale: 200 µm. **B**: HE staining of paraffin embedded cell line UKF-PEC1; scale: 50 µm. **C**: HE staining of paraffin embedded cell line UKF-PEC1^r^5-FU^500^; scale: 50 µm. **D**: HE staining of paraffin embedded cell line UKF-PEC1^r^CDDP^2500^; scale: 50 µm. **E**: HE staining of paraffin embedded cell line UKF-PEC1^r^CDDP^2500^/^r^5-FU^500^; scale: 50 µm

Morphology of the primary tumor, the parental cell line and the drug-resistant sublines after paraffin embedding and HE staining are presented in Fig. 2. In addition, viability assays against paclitaxel, docetaxel, olaparib, and rucaparib were determined for the cell lines UKF-PEC1, UKF-PEC1^r^5-FU^500^, UKF-PEC1^r^CDDP^2500^, and UKF-PEC1^r^CDDP^2500^/^r^5-FU^500^, as well as UKF-PEC3 and are presented in Table 1.

### Sequencing of the primary tumor yielded suboptimal reads

The sequencing of the FFPE cell line samples shows sufficient DNA sequencing quality with a respective value > 90. In addition, all RNA samples have a value > 500,000, indicating sufficient RNA sequencing quality. However, the sample from the primary tumor, embedded 2014, shows limited DNA sequencing quality. A quality control of the DNA using TapeStation showed a markedly lower DIN value of 1.5 compared to the cell line samples, which can lead to reduced sequencing quality. Repeating the analysis with another tumor block did not result in better sequencing outcome. Quality parameters are summarized in Supplementary Table 1.

### Sequencing results

#### Analysis of amplifications

We could show amplifications of TERT and RICTOR in the UKF-PEC cell line. In the 5FU-adapted subline, we detected amplifications of TERT, RICTOR and CDKN2B. In the CDDP adapted subline, MRE11, RAD51B and AKT1 showed amplifications. The cell line adapted to CDDP and 5-FU showed a PTEN amplification (Table 2).

**Table 2:** Frequency of detected amplifications/copy number variations in the cell lines

Genes	UKF-PEC1	UKF-PEC1^r^5-FU^500^	UKF-PEC1^r^CDDP^2500^	UKF-PEC1^r^CDDP^2500/ r^ /5-FU^500^
TERT	8.10	10.40	-	-
RICTOR	11.64	18.62	-	-
CDKN2B	-	5.18	-	-
PTEN	-	-	-	6.06
MRE11	-	-	4.74	-
RAD51B	-	-	4.78	-
AKT1	-	-	5.52	-

Mutations are reported when frequency exceeds 5

#### Analysis of mutations

The mutation analysis showed mutations of CDKN2A, FGFR4, NOTCH3, RAF1, RB1, SLX4, TERT, and TP53 in UKF-PEC1.

In the 5-FU-adapted subline, also mutations of CDKN2A, FGFR4, NOTCH3, RAF1, RB1, SLX4, and TP53 were found. Additionally, MLH1 was mutated in the 5-FU-radapted subline. In contrast to the parenteral cell line, TERT was not mutated.

The CDDP-resistant subline had also the above-mentioned mutations of the parental UKF-PEC1 cells. The CDDP-resistant subline harbored additional mutations in CREBBP, FGF3, NTRK2, PIK3CB, and RAD50 (Table 3).

**Table 3:** Frequency of detected point mutations in the cell lines

Genes	UKF-PEC1	UKF-PEC1^r^5-FU^500^	UKF-PEC1^r^CDDP^2500^	UKF-PEC1^r^CDDP^2500 r^ 5-FU^500^	Assessment^a^
*BRCA2*	-	-	-	4,39**	3
*CDK12*	4,84**	-	-	-	3
*CDKN2A*	98.86	98.85	97.72	98.45	1
*CREBBP*	-	-	45.1	-	3
*FGF3*	-	-	14.58	-	3
*FGFR4*	41.01	47.51	53.67	69.15	5
*MLH1*	-	5.66	-	-	5
*NOTCH3*	34.88	43.88	23.28	30.59	3
*NTRK2*	-	-	17.51	-	3
*PIK3CA*	-	-	4,3**	-	3
*PIK3CB*	-	-	17.43	-	3
*RAD50*	-	-	16.05	-	3
*RAF1*	66.73	76.85	56.45	66.7	1
*RB1*	100	100	100	97.64	3.4
*SLX4*	65.16	36.1	74.16	60.89	3
*TERT*	86.66	-	-	-	3.5
*TP53*	38.26	33.75	82.3	97.05	1
*TSPAN31*	-	3**	-	-	3

^a^Pathogenic = 1, likely pathogenic = 2, VUS = 3, benign = 4, SNP = 5

Mutations are reported when frequency exceeds 5 (**or close to 5)

#### HRD in the primary tumor and the cell lines

**Fig. 3:**
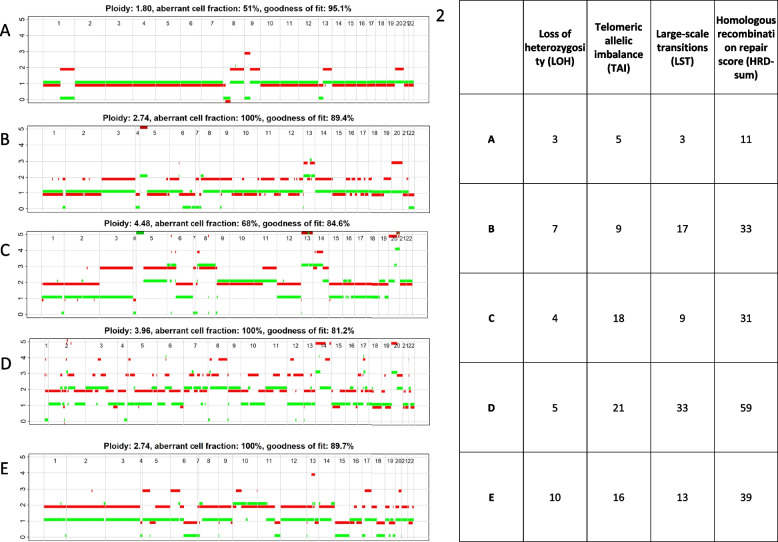
1. ASCAT Profiles: **A**: Primary tumor, **B**: UKF-PEC1, C: UKF-PEC1^r^5-FU^500^, D: UKF-PEC1^r^CDDP^2500^, E: UKF-PEC1^r^CDDP^2500^/^r^5-FU^500^ 2. Homologous recombination repair scores for the cell lines **A**: Primary tumor, **B**: UKF-PEC1, **C**: UKF-PEC1^r^5-FU^500^, **D**: UKF-PEC1^r^CDDP^2500^, **E**: UKF-PEC1^r^CDDP^2500^/^r^5-FU^500^ as the sum of loss of heterozygosity, telomeric allelic imbalance and large-scale transitions

**Fig. 4:**
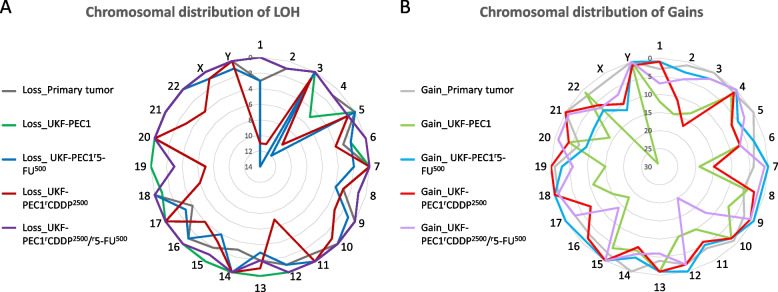
**A**: Chromosomal distribution of LOH. **B**: Chromosomal distribution of Gains

We found an increased HRD score in the subline adapted to CDDP. The cell lines adapted to 5-FU and the cell line with resistance against CDDP and 5-FU displayed lower HRD scores. In the cell line adapted with CDDP large scale transitions are remarkably more common than in the other cell lines. HRD scores and ASCAT profiles are displayed in Fig. 3. Detailed results of chromosomal gains and losses of the cell lines and the primary tumor are presented in Fig. 4.

#### Immunohistochemistry

**Fig. 5:**
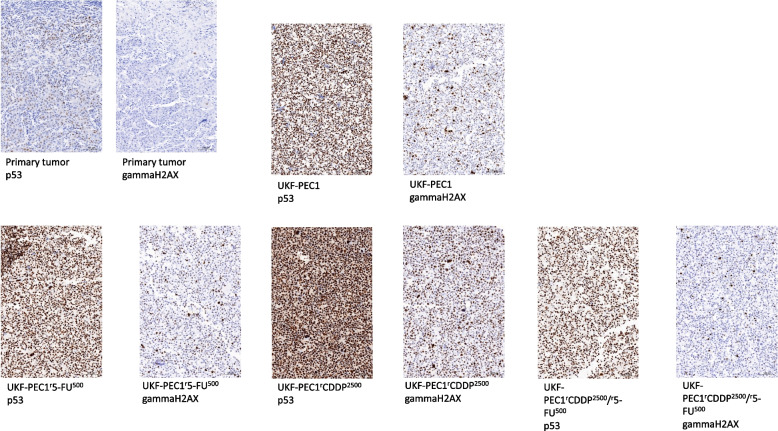
Staining results for p53 and gammaH2AX in the primary tumor and the cell lines UKF-PEC1, C: UKF-PEC1^r^5-FU^500^, D: UKF-PEC1^r^CDDP^2500^, E: UKF-PEC1^r^CDDP^2500^/^r^5-FU.^500^20 × digitized, 10 × magnification

Staining of the primary tumor and the cell lines for p53 and gammaH2AX was performed. The cell lines showed p53 overexpression, whereas the primary tumor showed a p53 wildtype pattern. For gammaH2AX no indication for DNA double-strand breaks were noted in the primary tumor. All cell lines showed occurrence of DNA double-strand breaks. Remarkably, the cell line adapted to CDDP showed an accumulation of DNA double-strand breaks in line with the increased HRD score. The staining results are summarized in Fig. 5.

## Discussion

In metastasized penile cancer, systemic therapy is the only treatment option left, but prognosis in this stage remains poor. Treatment failure at this stage is commonly caused by the development of resistance to chemotherapy. Therefore, efficient chemotherapies or targeted therapies are urgently needed. Penile cancer cell line models are essential tools to study the molecular mechanisms underlying tumor progression and resistance to chemotherapy [19, 29, 30]. Here, we have successfully established a penile cancer cell line including a set of sublines with acquired resistance to the frequently used chemotherapeutic agents 5-FU and CDDP [7–9]. The 5-FU-adapted penile cancer subline displayed cross-resistance to CDDP. Cross-resistance of 5-FU-adapted penile cancer cells to CDDP has not previously been reported.

Mutations detected in all cell lines were: CDKN2A, FGFR4, NOTCH3, RAF1, RB1, SLX4, and TP53. The genes CDKN2A, RAF1, RB1, and TP53 are part of the p16-cyclin D-CDK4/6-retinoblastoma protein (RB1) pathway (CDK4 pathway). It promotes G1–S cell-cycle transition and is often defective in cancer and, therefore, dysregulation of CDKs are a hallmark of cancer [31].

RAF1 is part of the MAPK signaling pathway. Copy number alterations were described in penile carcinomas [13]. Rb1, CDKN2A and TP53 mutations are known to be involved in penile cancer development [32–34]. Genetic alterations and nucleotide changes in coding regions like TP53 and CDKN2A have been described as main cancer driver events, both in HPV-positive and in HPV-negative tumors [35, 36].

NOTCH3 is expressed predominantly in vascular smooth muscle, central nervous system, some thymocyte subsets and regulatory T cells as well as by resting macrophages. NOTCH3 expression and signaling plays a role in pro-inflammatory macrophage activation and in the activation of NF-κB [37]. It is important for cell proliferation, differentiation and apoptosis. The NOTCH pathway is known to be frequently altered in penile cancer samples [38]. In our study, NOTCH3 was mutated in all cell lines. A link between penile squamous cell carcinomas and NOTCH3 mutation has not been described so far.

We found FGFR4 mutations in all our penile cancer cell lines. FGFR4 belongs to the family of fibroblast growth factor receptors. FGFR4 mutations were described in many tumors, i.e. breast, colon, and head and neck with controversial interpretation of their significance [39]. FGFR4 alterations were also described in penile squamous cell carcinoma cell lines [13].

SLX4 coordinates three different endonucleases directing cleavage and resolution of damaged DNA and Holliday junctions arising between homologous DNA duplexes [23, 40]. We have seen SLX4 mutations in all of the examined cell lines. As of today, it is estimated that SLX4 is essential in cancer [41–44] but SLX4 mutations in penile carcinomas were not described by the time of writing.

CDK12 is a well-known gene that is involved in carcinogenesis [45, 46]. Interestingly, CDK12 mutations were only found in the parental UKF-PEC1 cell lines, while sublines with acquired resistance to chemotherapy did not show these mutations. According to mycancer genome, CDK12 mutations are discovered in less than 5% of penile carcinoma patients investigated [47].

It was shown in this study, that amplifications, copy number variations and mutations in the cell lines adapted to CDDP and 5-FU are more in number than in the double resistant cell line. The cell line UKF-PEC1^r^CDDP^2500^/^r^5-FU^500^ was adapted with both substances simultaneously. The cell lines UKF-PEC1^r^5-FU^500^ and UKF-PEC1^r^CDDP^2500^ were adapted individually. If the same mutations were seen in each case, this would require that the cells always react in the same way when adapted to a cytostatic drug. However, the cells do not do this, but can develop different mechanisms as shown before [40, 48, 49].

To prove p53 expression in the primary tumor and the cell lines, immunohistochemical staining for p53 was performed. Sequencing results showed low level p53 mutations (c.524G > A with an allele frequency of 11% and a low coverage of 305 as well as c.574C > T with an allele frequency below 5%) in the primary tumor. According to the sequencing results the cell lines harbored the following p53 mutations: UKF-PEC1 harbored c.574C > T, UKF-PEC1^r^5-FU^500^ harbored c.574C > T, UKF- UKF-PEC1^r^CDDP^2500^ harbored c.524C > A and UKF-PEC1^r^CDDP^2500^/^r^5-FU^500^ harbored c.524C > A mutations. Consequently, the primary tumor showed a p53 wild type pattern via immunohistochemistry and the cell lines showed strong staining results. The antibody applied here reacts with wild type and mutant type of the p53 protein. Nevertheless, different p53 antibodies are present which can react with specific mutations of the p53 gene.

Additionally, staining for gammaH2AX was performed. GammaH2AX is a marker which is indicative for the occurrence of double strand breaks and is linked to genomic instability. It was studied in a number of cancer subtypes [50]. Cisplatin, as a chemotherapeutic drug, is linked to DNA crosslinking and stimulates H2AX phosphorylation [51]. Immunohistochemically a strong staining was observed in the cell lines compared to the primary tumor indicative of DNA double-strand breaks.

### 5-FU Resistance

Immunohistochemistry for defective mismatch repair (MMR) proteins like MLH1, MSH2, MSH6, and PMS2 was analyzed by Stoehr et al. in 70 patients with penile cancer. They showed a normal expression of the MMR proteins [52]. In contrast to that, our study showed mutations of MLH1 in the 5-FU-resistant subline. According to mycancer genome (www.mycancergenome.org), MLH1 is altered in 3.85% of penile carcinoma patients [47]. CDKN2B was amplified in the cell line resistant to 5-FU. That finding is interesting because loss of CDKN2B was described in penile carcinomas [53]. RICTOR was also amplified in the cell line resistant to 5-FU. RICTOR, as part of the mTOR signaling network, is indirectly involved in cell survival and actin cytoskeleton organization. Amplification and overexpression was noted in several cancer, e.g. lung cancer [54].

TERT, the telomerase reverse transcriptase gene, is found to be reactivated in many cancer cells and enables cancer cells to evade senescence resulting from telomere shortening [55]. TERT mutations were described in a high frequency of penile carcinomas, especially in the non-HPV related subtypes [55]. TERT was amplified in 5-FU-resistant cells. TERT amplifications have not been described in penile squamous cell carcinomas, so far.

### CDDP Resistance

It is well known that CDDP alters the expression of many genes involved in several cellular processes. Ryan et al. could show increase of sensitivity to CDDP in neuroblastoma cells by downregulation of NTRK2, a potent oncogene involved in chemotherapy-resistance in neuroblastoma, with mR204 [56]. In our study, NTRK2 was also mutated in the CDDP-resistant subline compared to the chemo-naïve cell line. NTRK2 and its fusions are of interest in different tumors, i.e. secretory carcinoma of the salivary gland and lung adenocarcinoma, because they can be targeted by specific inhibitors. Although NTRK2 mutations have been described, their contribution towards oncogenesis and therapy with NTRK inhibitors is not yet clear [57].

PIK3CA is a known oncogene involved in the PI3K-AKT-mTOR pathway which plays a role in many human cancers with respect to cellular proliferation, survival and angiogenesis. However, Adimonye et al. conclude, that this pathway is not a key driver in the carcinogenesis of penile squamous cell carcinomas [58]. Fallahi et al. concluded that several genes including PIK3CA and PIK3CB act as hub genes in the cisplatin-responsive regulatory network at the pro-apoptotic stages. In line with this study, we showed that PIK3CA and PIK3CB were frequently mutated [59]. PIK3CB mutations activate PI3K-dependent signaling, increase cancer cell proliferation and promote tumorigenic growth [60]. A link between penile carcinomas and PIK3CB mutations has not been delineated, yet by the time of writing.

CREBBP and FGF3 mutations have been frequently described to be associated with tumorigenesis [61–63]. CREBBP and FGF3 mutations have also been described in penile squamous cell carcinomas [33]. In our study, we could see an elevated mutation rate in the CDDP-resistant subline. However, it is not clear whether these mutations contribute to CDDP resistance.

The MRE11-RAD50-NBS1 protein complex is able to recognize and process DNA double strand breaks and maintains genomic stability in the cell. Mutations in the genes are associated with many cancers, i.e. colon and breast carcinoma [64]. Alblihy et al. showed that RAD50 deficiency is a predictor of platinum sensitivity in sporadic epithelial ovarian cancers. We could show a more frequently mutated RAD50 gene in the CDDP-resistant subline [65].

The CDDP-resistant subline showed further amplifications: MRE11, RAD51B and AKT1. MRE11 and RAD51B play a role in the DNA repair reactions through homologous recombination [66]. An association with breast and ovarian cancer predisposition was shown so far [67]. AKT1 amplification was shown to be associated with CDDP resistance in human lung cancer cells [68]. Copy number alterations of AKT1 were described in penile carcinomas [32].

### HRD in penile squamous cell carcinomas

The HRD score quantifies the so-called genomic scar evoked by HRD and has become a diagnostic marker [69]. HRD failure is frequently observed in solid tumors and is a marker indicating sensitivity to poly(ADP-ribose) polymerase (PARP) inhibitor therapy [70]. HRD was also shown to be associated with patient survival and chemotherapy response [69]. Yang et al. also showed, that a high HRD score is associated with an immune-sensitive microenvironment in HRD related cancer types [69]. For penile carcinomas, tumors have been described with different immune cell infiltrations [71]. Therefore, data comparing the immune microenvironment and HRD score in penile neoplasia could aid in decision making for precision medicine in the orphan disease. Because data on penile carcinomas is sparse, more cases need to be evaluated in that context. In our case, a high HRD score was detected in the sublines adapted to growth in the presence of CDDP. That is in concordance with data from literature describing impaired DNA repair mechanisms following administration of chemotherapy [70]. Additionally, a gene fusion PCNX-RAD51B was shown in this cell line, being in line with the finding of DNA damage repair pathway association. This fusion was described before in a case of cervical carcinoma [72]. Next to the method applied here, there are commercially available test kits interrogating different sets of genes to determine a HRD score (reviewed in [70]). Determining the HRD score needs testing and validation in different tumor entities, for example in prostate cancer due to different mutational landscape in contrast to ovarian cancer [73]. In our cohort a combinatory chemotherapy resulted in decreased HRD score compared to CDDP monotherapy. This needs to be proven on a bigger cohort. Therefore, our model can contribute to gaining data on HRD landscape in penile carcinomas since data on HRD are sparce in the disease. In a publication testing 33 cancer types within the TCGA data set, a cut off of 42 was also applied [74]. By the time of writing, to the best of our knowledge no TCGA data existed describing HRD scores in penile carcinomas. The increased HRD score observed in UKF-PEC-1^r^CDDP^2500^ cells was not associated with enhanced sensitivity to the taxanes docetaxel and paclitaxel and the PARP inhibitors olaparib and rucaparib, indicating that an increased HRD score is not necessarily associated with elevated PARP inhibitor sensitivity. This may be due to molecular changes associated with resistance formation to chemotherapeutic agents such as increased levels of membrane transporters, e.g. ABCB1, or acquired resistance to apoptosis, which may also provide resistance to PARP inhibitors [75, 76]. Immunohistochemical staining supports the high HRD score found here with accumulation of double strand breaks as tested with gammaH2AX.

### Study limitations

We performed an OCA panel on one case of penile carcinoma with additional chemo-resistant sublines. Therefore, further samples need investigation to confirm the mutational status. Additionally, and due to the nature of the test, only limited numbers of genes were targeted. The primary tumor paraffin block material aged seven years in the archive prior to investigation whereas the cell lines were paraffin embedded in the same year as the processing procedure took place. Therefore, impaired DNA and RNA quality of the primary tumor can be explained.

Additionally, the HRD score of 42 needs validation in bigger cohorts of penile carcinomas to establish an individualized score for this entity.

## Conclusion

We generated a p16 negative cell line of a squamous cell carcinoma of the penis, and from this parental cell line three chemo-resistant sublines were established: one subline adapted to 500 ng/ml 5-FU, one subline adapted to 2500 ng/ml CDDP and one subline adapted to both drugs. A molecular profile including the HRD score was performed with this panel of cell lines. Acquired chemoresistance to CDDP resulted in an increased HRD score. With the use of PARP inhibitors, an increased sensitivity to these drugs was not shown in our cell line model.

## Supplementary Information

**Additional file 1: Supplementary Figure 1.** Dose-response curves for UKF-PeC1, UKF-PeC3 and their adapted chemoresistant sublines against CDDP and 5-FU.

**Additional file 2: Supplementary Table 1.** Quality parameters for the primary tumor and the cell lines investigated.

## Data Availability

The datasets generated and/or analysed during the current study are available under BioProject ID PRJNA904558.
