# Supplementary material for: Increased HRD score in cisplatin resistant penile cancer cells
Source: BMC Cancer. 2022 Dec 23;22:1352. doi: 10.1186/s12885-022-10432-7 (PMC9789628; doi:10.1186/s12885-022-10432-7)
Supplement: Supplementary file 1 — Additional file 1: Supplementary Figure 1. Dose-response curves for UKF-PeC1, UKF-PeC3 and their adapted chemoresistant sublines against CDDP and 5-FU. [file 12885_2022_10432_MOESM1_ESM.pptx]

## Slide 1
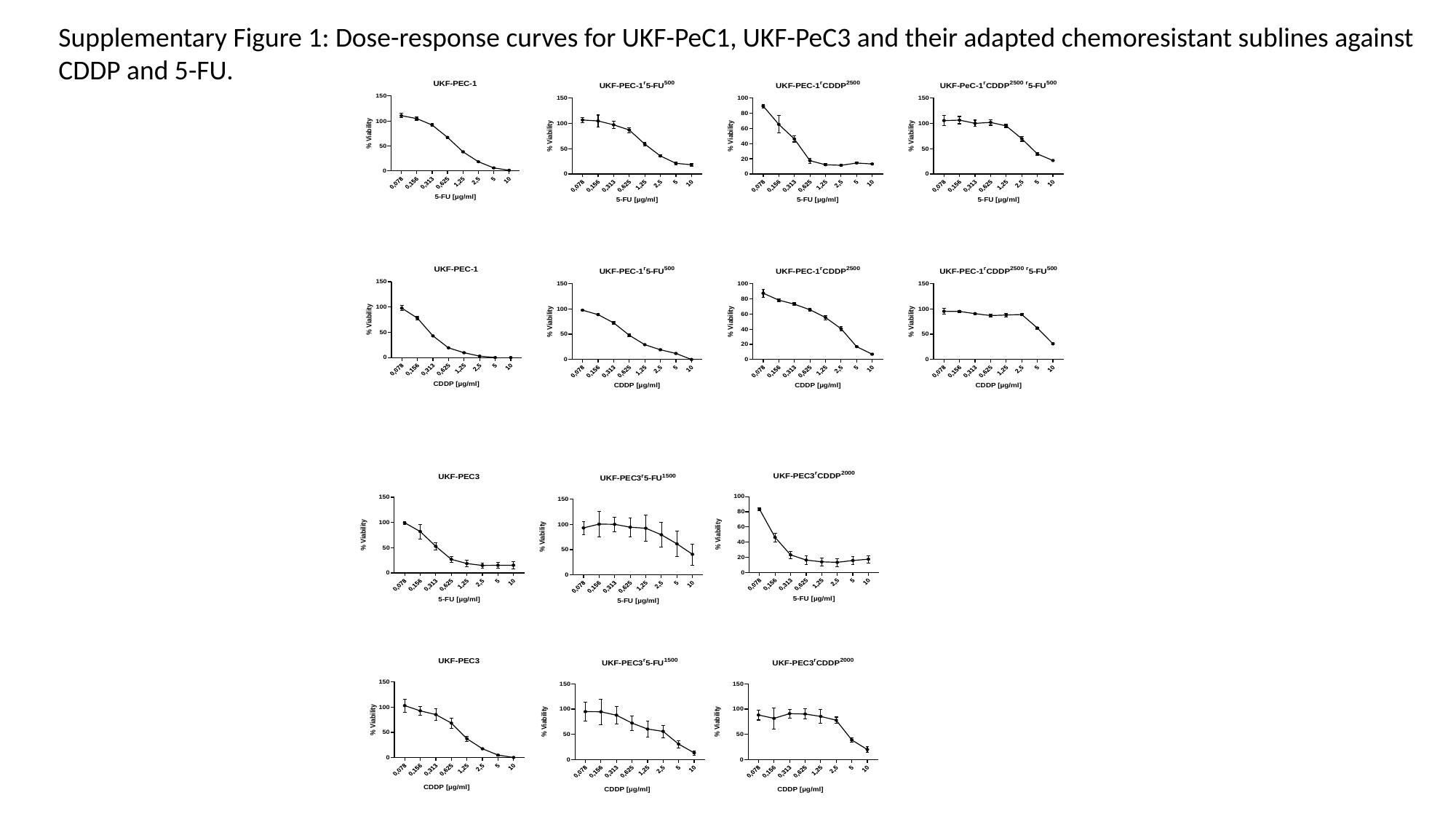

Supplementary Figure 1: Dose-response curves for UKF-PeC1, UKF-PeC3 and their adapted chemoresistant sublines against CDDP and 5-FU.
